# Relationships among body mass, brain size, gut length, and blood tryptophan and serotonin in young wild-type mice

**DOI:** 10.1186/1472-6793-9-4

**Published:** 2009-03-25

**Authors:** Ricardo Albay, Angela Chen, George M Anderson, Maggie Tatevosyan, Skirmantas Janušonis

**Affiliations:** 1 Department of Psychology, University of California, Santa Barbara, California, USA; 2 Yale University Child Study Center, New Haven, Connecticut, USA

## Abstract

**Background:**

The blood hyperserotonemia of autism is one of the most consistent biological findings in autism research, but its causes remain unclear. A major difficulty in understanding this phenomenon is the lack of information on fundamental interactions among the developing brain, gut, and blood in the mammalian body. We therefore investigated relationships among the body mass, the brain mass, the volume of the hippocampal complex, the gut length, and the whole-blood levels of tryptophan and 5-hydroxytryptamine (5-HT, serotonin) in young, sexually immature wild-type mice.

**Results:**

Three-dimensional reconstructions of the hippocampal complex were obtained from serial, Nissl-stained sections and the gut was allowed to attain its maximal relaxed length prior to measurements. The tryptophan and 5-HT concentrations in the blood were assessed with high-performance liquid chromatography (HPLC) and the sex of mice was confirmed by genotyping. Statistical analysis yielded information about correlative relationships among all studied variables. It revealed a strong negative correlation between blood 5-HT concentration and body mass and a strong negative correlation between the brain mass/body mass ratio and gut length. Also, a negative correlation was found between the volume of the hippocampal complex and blood tryptophan concentration.

**Conclusion:**

The study provides information on the covariance structure of several central and peripheral variables related to the body serotonin systems. In particular, the results indicate that body mass should be included as a covariate in studies on platelet 5-HT levels and they also suggest a link between brain growth and gut length.

## Background

The present study was motivated by the platelet hyperserotonemia of autism, a long-standing problem in autism research. It is an extension of our previous experimental and theoretical investigations [1-3] that suggest that the platelet hyperserotonemia is inherently a systems problem in that it requires understanding relationships among various components of the peripheral serotonin (5-hydroxytryptamine, 5-HT) system and the developing brain.

The platelet hyperserotonemia of autism is defined as elevated 5-HT levels in the blood platelets of individuals diagnosed with autism spectrum disorders (ASDs) [4-6]. To date, this finding has been replicated by many studies in ethnically diverse autistic groups [7-15]. While a number of genes may contribute to ASD risk [16-21], the replicability of the platelet hyperserotonemia suggests that these genes may affect a small set of biological networks [3,22].

The 5-HT sequestered in platelets is synthesized from tryptophan by enterochromaffin cells in the gut wall [23], with negligible contribution from serotonergic neurons of the enteric nervous system [24]. Some of the gut 5-HT diffuses into the systemic blood circulation, where most of it is rapidly cleared by the liver and lungs [25,26]. The remaining free 5-HT in the blood plasma can be taken up by platelets that express the serotonin transporter (SERT). Early studies have suggested that the platelet hyperserotonemia of autism may be caused by altered function of SERT in blood platelets [27]. More recent research has shown that SERT polymorphic variants do partially determine platelet 5-HT uptake rates [28], but that these polymorphisms, alone, do not cause the platelet hyperserotonemia of autism [28,29] or contribute to autism itself [10,30-33]. Other studies have suggested that the platelet hyperserotonemia may be caused by altered 5-HT production in the gut [1,34-37], but current experimental evidence remains inconclusive. Also, no link between platelet hyperserotonemia and increased intestinal permeability has been found in children with pervasive developmental disorders [38].

Several studies have recently proposed that the platelet hyperserotonemia of autism may be caused by interactions among several components of the peripheral 5-HT system [2,14,37,39]. Specifically, the amount of 5-HT stored in blood platelets may depend on the dynamic interplay among the gastrointestinal system, blood circulation, vascular beds, and platelets themselves [2]. Understanding these interactions in the peripheral 5-HT system may lead to new insights into the normal function of the central nervous system. Tryptophan, the 5-HT precursor, is actively transported across the blood-brain barrier (BBB) and, while 5-HT is generally thought to not cross the BBB, some transfer may occur at elevated 5-HT concentrations [40]. Brain neurons express the same SERT as platelets [41], and SERT is also expressed by brain endothelial cells [42,43]. Also, brain and enteric neurons express many of the same serotonin receptors [44].

Our previous theoretical investigation has shown that platelet 5-HT levels may be a function of a number of parameters and their interactions, among them the volume of the circulating blood and the length of the gut [2]. Based on these previous findings, we investigated correlative relationships among several key components of the peripheral 5-HT system and the brain in young, sexually immature mice. Specifically, we measured the body mass, the brain mass, the volume of the hippocampal complex, the relaxed gut length, and the whole-blood 5-HT and tryptophan levels in the same individual wild-type mice at two weeks after birth. In this study, the hippocampal complex served as a convenient brain structure the borders of which are clearly defined in Nissl-stained sections. The hippocampal complex may also play a role in ASDs [45-47].

The principal aim of the present study was to reveal the system's covariance structure [48] which can guide experimental design and can be used in advanced, causal modeling. Therefore, strong emphasis was placed on statistical power in an effort to obtain reasonably accurate correlation coefficients, rather than merely significant or non-significant results [49]. One of the advantages of this approach is that the obtained covariance matrix can be combined with results of future studies and used in structural equation modeling, a powerful modeling technique for complex systems.

The focus of the present study was restricted to a postnatal developmental time that may offer insights into central and peripheral alterations in ASDs. We have previously shown that, by two weeks after birth, 5-HT_1A _receptor-knockout mice develop platelet hyperserotonemia [1]. Studies in rats suggest that at this time the development of the hippocampus and the cerebellum are strongly controlled by the state of the brain serotonin system. In the first two postnatal weeks, the expression of 5-HT_1A _receptors increases in the forebrain, but strongly decreases in the cerebellum [50]. Between postnatal days 10 and 21, the expression of 5-HT_1A _receptors in the pyramidal cells of the hippocampus shifts from somatic to dendritic and, between postnatal days 10 and 16, the expression of 5-HT_1A _receptors in S100-positive hippocampal astrocytes declines from 90% to 20% [51]. The binding of 5-HT_4 _receptors in the globus pallidus and the substantia nigra sharply increases between postnatal days 9 and 12 and then strongly decreases between postnatal days 12 and 21 [52]. Even though little is known about whether similar changes occur in the human brain, they appear likely since the 5-HT_1A _expression in the human cerebellum shifts from high in the neonatal stage to very low in adulthood [53] resembling the developmental pattern of 5-HT_1A _receptor expression in rats [50].

## Methods

### Animals

Timed-pregnant, wild-type CD-1 mice were purchased from Charles River Laboratories, Inc. (Wilmington, MA). They arrived at 15–17 days of gestation and were housed in the UCSB animal facility in individual cages on a 12:12 light-dark cycle (lights on at 07:00, off at 19:00). Mice were checked every morning (before 12:00) and the delivery day was considered to be postnatal day (PD) 0. The pups were kept with the dam until used. Pups from six litters were analyzed. All litters were normal size (12–14 pups). All experiments were approved by the UCSB Institutional Animal Care and Use Committee.

### Tissue harvesting

A total of 63 pups from 6 litters were used in the study. At postnatal day 14 (5 litters) or 15 (1 litter), individual pups were removed from the cage, weighed, and immediately decapitated. The trunk blood was collected into a 1.5 mL microcentrifuge tube containing 25 μL of 5% Na_2_EDTA dissolved in water, as previously described [1]. The tube was immediately pulse-vortexed at low speed and kept at room temperature until the dissections of the brain and gut were completed. Following the dissections, the blood sample was visually examined for blood clots, the volume of the collected blood was measured, and the sample was stored in 20 μL aliquots at -75°C. The brain was dissected out of the skull with a fine rongeur, immediately weighed in phosphate-buffered saline (0.1 M PBS, pH 7.2) on a precision balance, immersion-fixed overnight in 4% paraformaldehyde in PBS at 4°C, allowed to sink in 30% sucrose in PBS at 4°C, and processed as described below. The gastrointestinal tract (the stomach through the rectum) was dissected out of the trunk and allowed to relax in PBS at 4°C for 10–13 days before photographed. The distal tail was stored at -75°C for sex-genotyping. All analyses were done blindly with respect to the values of the other variables in the same individual mice.

### Brain

Following cryoprotection, 17 brains (from 2 litters) were embedded in 20% gelatin (type A; 275 bloom), immersed in formalin with 20% sucrose for 3 hours at room temperature, and sectioned coronally on a freezing microtome at 50 μm thickness into PBS in 96-well trays. Every lost section was recorded and the percentage of missing sections was not allowed to exceed 9%. The sections were immediately mounted onto glass slides coated with 0.5% gelatin and 0.05% chromium potassium sulfate, allowed to air-dry, Nissl-stained with 0.25% thionin, dehydrated in a graded series of ethanols, cleared in Xylenes, and coverslipped with Permount. Since fixation and embedding may alter tissue volume [54,55], care was taken to ensure all brains and sections were exposed to the reagents for the same length of time.

**Figure 1:**
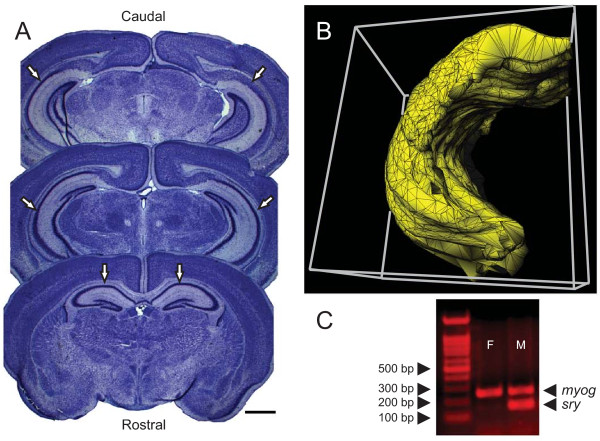
**Three-dimensional reconstruction of the hippocampal complex and sex-genotyping**. (**A**) Representative Nissl-stained sections through the mouse brain used in three-dimensional reconstructions of the hippocampal complex. Scale bar is 1 cm. (**B**) A three-dimensional reconstruction of the hippocampal complex. (**C**) The *sry *and *myog *(control) bands in female (F) and male (M) pups detected by sex-genotyping.

Images of serial sections were captured with a Zeiss Axio Imager Z1 equipped with a color digital camera (AxioCam HRC Rev. 2) using a 1× objective (Fig. 1A), imported into the *Reconstruct *software (version 1.0.9.6; ), and the outline of the left hippocampal complex was traced from the most rostral section containing the pyramidal cells to the most caudal section containing the dental gyrus (Figs. 39–64 of [56]). In this rostro-caudal block, the traced regions included the hippocampus proper, the fimbria, the dentate gyrus, the presubiculum, and the subiculum. If a section was missing, the thickness of each of the two neighboring sections was digitally increased by 50%. The quality of tracing was assessed by rotating the 3D-reconstruction and visually inspecting it for discontinuities (Fig. 1B). No digital smoothing was used.

#### Guts

**Figure 2:**
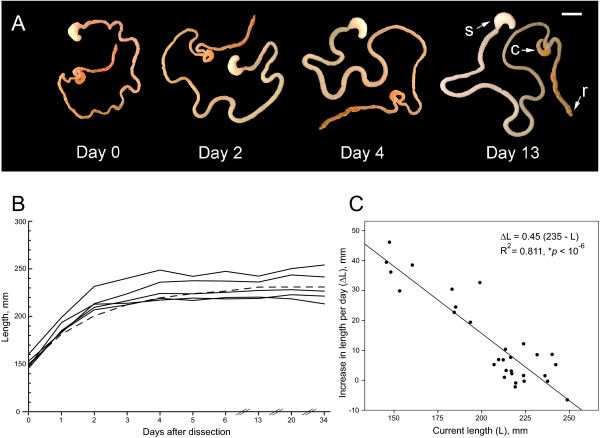
**Gut relaxation**. (**A**) The relaxation of the same representative gastrointestinal tract kept in PBS at 4°C and photographed at 0, 2, 4, and 13 days after dissection. Abbreviations: s, stomach; c, coecum; r, rectum; scale bar is 1 cm. (**B**) The relaxation of five guts (measured from the pylorus of the stomach to the coecum) kept in PBS at 4°C and measured over the course 34 days. The dashed line represents the theoretical relaxation (Eq. 2) of a gut with the initial length of 150 mm. (**C**) The relationship between the current gut length and the increase in gut length per day. The data points were obtained from the five guts used in (b); for better precision of the linear regression (line), only the initial 6 days were included in the analysis. The rate of relaxation was calculated as the absolute difference in length between the current day and the next day.

To our knowledge, there are no standard protocols for measuring gut length. Since the length of the gut increases considerably after dissection (probably due to muscle relaxation), we investigated this process experimentally (see Results; Fig. 2) and developed a protocol that allowed measuring gut length with high reliability. Following 10–13 days of relaxation in 0.1 M PBS (pH 7.2) at 4°C, gastrointestinal tracts were digitally macro-photographed (Fig. 2A) in 10 cm Petri dishes with shallow PBS (in order to avoid vertical loops). A millimeter-ruler was included in the images for scale. The images were imported into *Reconstruct*, and the length of the gut was determined by tracing both sides of the gut from the pylorus of the stomach to the coecum and averaging the length of the two traces. The remaining part of the gut was not measured because it was difficult to control if the dissection included the most distal part of the gastrointestinal tract. This approach produced consistent measures over many days (Fig. 2B) and was superior to obtaining only one trace equidistant from the two sides of the gut.

### Whole-blood tryptophan and 5-HT

The concentration of tryptophan and 5-HT in the blood samples was measured by high-performance liquid chromatography (HPLC) as previously described [1]. Blood samples were prepared by addition of 100 μL of 25% ascorbic acid, 100 μL of 5% sodium metabisulfite, 10 μL of 10 ng/μL *N*-methylserotonin (NMS, internal standard) to frozen whole blood samples. After thawing and mixing, 75 μl of 3.4 M perchloric acid was added, the samples were kept on ice for 10 minutes, centrifuged, and the supernate was stored at -80°C until analyzed. Analysis was performed by HPLC with fluorometric detection: a 25 × .46 Altex Ultrasphere column heated to 40°C was eluted with a mobile phase consisting of 70% 0.1 M NaH_2_PO_4 _(pH 4.7) containing 150 mg/L octyl sulfate, 20% methanol. Compounds were detected with a Shimadzu RF 10-AXL fluorometer, with excitation and emission wavelengths of 285 and 345 nm, respectively, quantitated by comparing peak heights ratios (analyte/NMS) to those observed for standards, and concentrations expressed as ng per mL. The neurochemicals were determined with typical intra- and inter-assay coefficients of variation of less than 5% and 10%, respectively. In the final calculations, the dilution of the collected blood in the Na_2_EDTA solution was factored out; therefore, the reported values represent tryptophan and 5-HT concentrations in the undiluted whole blood.

### Genotyping

Since sexing of young mice based on the appearance of their external genitalia is difficult, the anogenital distance can be used as a more reliable measure. In the present study, several organs had to be rapidly extracted destroying the body in the process. Therefore, in order to avoid ambiguity and human errors, mice were sexed by genotyping. This approach also allows permanent storage of the extracted DNA which can be used for future analyses. Genomic DNA was isolated with the DNA Isolation Kit (Lamda, St. Louis, MO). Briefly, 1 cm of the tail was digested in a lysis buffer at 56°C overnight, the DNA was precipitated with ethanol, dissolved in 150 μL of Tris-EDTA buffer (TE, pH 8.0), and stored at -20°C. The *sry *region located on the Y chromosome was amplified by polymerase chain reaction (PCR) using the HotStarTaq Master Mix Kit (Qiagen, Valencia, CA). The *sry *primers were 5'-AACAACTGGGCTTTGCACATTG-3' (forward) and 5'-GTTTATCAGGGTTTCTCTCTAGC-3' (reverse), and the control (*myog*) primers [57] were 5'-TTACGTCCATCGTGGACAGC-3' (forward), 5'-TGGGCTGGGTGTTAGTCTTA-3' (reverse). The PCR reaction mixture (50 μL) contained 25 μL of the Master Mix, 1 μL (0.5 μM) of each primer, 0.5 μL of the DNA sample, and 20.5 μL water. The amplification was performed in a PxE thermal cycler (ThermoFisher Scientific) with the following conditions: initial activation at 95°C for 15 min, 33 cycles of amplification (94°C for 40 sec; 60°C for 40 sec; 72°C for 1 min), and final extension at 72°C for 9 min. The PCR products were run at 120 V on 1.5% agarose gel in Tris-acetate-EDTA buffer (TAE) containing 5% ethidium bromide and digitally imaged with a DigiDoc-It UV transillumination system (UVP, Upland, CA). The amplicon sizes were 146 bp/166 bp (*sry*, doublet) and 245 bp (*myog*) (Fig. 1C).

### Statistical analysis

Statistical analysis was carried out in SPSS 16.0.2 (SPSS Inc., Chicago, IL). Conceptually, the analysis was based on the general linear model (GLM) that considers correlation, ANOVA and ANCOVA to be special cases of linear regression [58]. Residuals were tested for normality with the Shapiro-Wilk test (with the level of significance of *p *< 0.05) and for independence with the Durbin-Watson test (DW; independence was assumed if the DW value was in the 1–3 range).

Unless otherwise noted, the forced-entry method was used in linear regression. A non-linear relationship between two variables was considered to be linear "in the parameters" [58], which allowed using standard GLM methods. In backward (stepwise) regression, Fisher's *F *critical value for variable removal was set at the standard significance level of 0.1. Pearson's correlation between two variables was used if the residuals of the linear regression between these two variables were normally distributed (otherwise, the Mann-Whitney test was used). The emphasis on the normality of residuals rather than of variables themselves follows the standard approach in GLM models. Specifically, it allows treating Student's independent *t*-test as a special case of Pearson's correlation between a continuous variable and a dichotomous variable (e.g., sex; Table 1).

**Table 1:** Differences between the sex groups and the cross-correlations between the variables

	**M** **(g)**	**BM** **(g)**	**BM/M**	**HC** **(mm^3^)**	**Trp** **(ng/mL)**	**5-HT** **(ng/mL)**	**5-HT/** **Trp**	**Gut** **(mm)**
**Sex**	F < M	F < M	F > M	F < M	F < M	F > M	F > M*	F > M
	**.185**	**.273**	**.325**	**.286**	**.584**	**.245**	**.040**	**.861**
	63	63	63	17	58	58	58	33
								
**M**		.768**	-.825**	.662**	-.089	-.594**	-.304*	.688**
		**< 10**^-6^	**< 10**^-6^	**.004**	**.507**	**10**^-6^	**.020**	**10**^-5^
		63	63	17	58	58	58	33
								
**BM**			-.165	.619**	.056	-.433**	-.262*	.400*
			**.196**	**.008**	**.676**	**.001**	**.047**	**.021**
			63	17	58	58	58	33
								
**BM/M**				-.571*	.229	.465**	.315*	-.745**
				**.017**	**.084**	**2·10**^-4^	**.016**	**10**^-6^
				17	58	58	58	33
								
**HC**					-.638**	-.505*	-.124	.427
					**.008**	**.046**	**.649**	**.146**
					16	16	16	13
								
**Trp**						.116	-.430**	-.293
						**.387**	**.001**	**.110**
						58	58	31
								
**5-HT**							.818**	-.264
							**< 10**^-6^	**.152**
							58	31
								
**5-HT/Trp**								-.004
								**.983**
								31
								
**N**	63	63	63	17	58	58	58	33
**Mean**	**6.511**	**.387**	**.061**	**8.127**	**16424**	**2069**	**.128**	**221.30**
**STD**	.954	.028	.008	1.054	2439	536	.037	11.25

Abbreviations: M, body mass; BM, brain mass; HC, volume of the hippocampal complex; Trp, blood tryptophan concentration; 5-HT, blood 5-HT concentration; Gut, length of the relaxed gut; N, total number of cases; STD, standard deviation. The two sex groups were compared using the exact Mann-Whitney test if the variable was BM/M or 5-HT/Trp; otherwise, Student's independent *t*-test was used. Spearman's correlation was used if either of the two non-dichotomous variables was BM/M or 5-HT/Trp; otherwise, Pearson's correlation was used. In the *sex *row, the "greater than" ("less than") sign indicates that the mean of the female group was greater (less) than the mean of the male group. In all other cells, the first number is the value of the correlation coefficient. In all cells, the second (bold) number is the significance of the corresponding test. In all cells, the third number is the total number of cases used in the test. The residuals of the linear regression with sex as the predictor variable and M or 5-HT as the dependent variable were not normally distributed (*p *= 6·10^-5 ^and 8·10^-6^, respectively). Therefore, the differences between the mean M and 5-HT values in the two sex groups were retested with the exact Mann-Whitney test, which confirmed they were not significant (*p *= 0.124 and 0.168, respectively).

## Results

### Data screening

The collected data (Additional file 1) for each of the variables were screened for quality blindly with respect to the values of the other variables. As described below, objective criteria were used to eliminate some cases from further analysis. Since in all reconstructed cases the percentage of missing sections in the hippocampal complex did not exceed 9%, all of the reconstructions were used. Cases with guts clearly damaged during dissection (e.g., split into more than two pieces) were not used in further analyses that included gut length as a variable. We investigated whether the presence of blood clots in some blood samples affected their tryptophan or 5-HT concentration. The mean tryptophan concentrations were not significantly different in the samples with and without blood clots (*t*(61) = 0.47, *p *= 0.644), but the mean 5-HT concentration was significantly lower in the former group (*t*(61) = 2.44, *p *= 0.018). Therefore, cases with blood clots (*N *= 5) were disregarded in all further analyses that included tryptophan or 5-HT as variables.

The collected blood volume varies from case to case and is strongly affected by the constriction of major blood vessels, the angle between the trunk and the collecting tube, and other factors. We therefore asked whether the amount of the collected blood correlated with the measured tryptophan and 5-HT concentrations (if all other parameters are equal and the measurements are accurate, no significant effect should be observed). The correlation between blood sample volume and blood 5-HT concentration was significant (*r *= -0.385, *p *= 0.003). However, the collected blood volume is also influenced by body size. Therefore, we calculated the partial correlation between blood sample volume and blood 5-HT concentration controlling for body mass and found that this correlation became non-significant (*r *= -0.085, *p *= 0.532). In contrast, the partial correlation between body mass and blood 5-HT concentration controlling for blood sample volume was highly significant (*r *= -0.495, *p *= 9·10^-5^). Therefore, all of these cases (*N *= 58) were used in further analyses that included tryptophan or 5-HT as variables. The relationship between body mass and blood 5-HT concentration was investigated further.

ANOVA showed significant litter effects on body mass (*F*_5,57 _= 40.3, *p *< 10^-6^), brain mass (*F*_5,57 _= 15.5, *p *< 10^-6^), hippocampal volume (*F*_1,15 _= 7.9, *p *= 0.013), blood tryptophan levels (*F*_5,52 _= 10.8, *p *< 10^-6^), blood 5-HT levels (*F*_5,52 _= 16.0, *p *< 10^-6^), and a near-significant litter effect on gut length (*F*_5,27 _= 2.54, *p *= 0.052).

### Gut relaxation

After dissection, the length of the gut increases over the course of several days until it reaches a plateau (Fig. 2A, B). We followed the relaxation of 5 guts in PBS at 4°C and plotted the rate of their length increase as a function of their current length (Fig. 2C). Linear regression revealed a strong relationship between these variables (*R*^2 ^= 0.811, *F*_1,28 _= 120.5, *p *< 10^-6^); the residuals were normally distributed (*p *= 0.18) and independent (DW = 2.45). This linear relationship suggests that gut relaxation can be modeled with the differential equation

(1) *dL/dt *= *k *(*γL*_0 _- *L*),

where *dL/dt *is the current length increase rate as a function of the current gut length (*L*), *L*_0 _is the initial gut length on the dissection day, *γL*_0 _is the final gut length (where *γ *> 1 is a constant independent of *L*_0_), and *k *is the length increase rate constant. Based on Eq. (1), we reanalyzed the gut relaxation data with linear regression using *dL/dt *as the dependent variable and *L*_0 _and *L *as the two predictor variables. Since according to Eq. (1) *dL/dt *= 0 when *L*_0 _= *L *= 0, the intercept of the regression line was fixed at the axis origin. The regression revealed that Eq. (1) well describes the relaxation process, since *L*_0 _and *L *explained virtually all variance in the length increase rate (*R*^2 ^= 0.947, *F*_2,28 _= 250.8, *p *< 10^-6^). The residuals were normally distributed (*p *= .993) and independent (DW = 1.72). The regression yielded *k *= 0.49 day^-1 ^and *γ *= 1.54. Since the solution of Eq. (1) is

(2) *L*(*t*) = *γL*_0 _+ *L*_0_*e*^-*kt *^(1 - *γ*),

plugging the obtained numerical *k *and *γ *values into Eq. (2) shows that, at 4°C, the gut reaches 95% of its final length (1.54 *L*_0_) in 4 days and 99% in 7 days. In the present study, we allowed guts to relax for 10–13 days; however, shorter relaxation times (6–8 days) should be sufficient to obtain accurate measurements.

### Cross-correlations

In the initial analysis, the residuals of the linear regression between each of the variable pairs were tested for normality. Of 7 pairs that failed the normality test (irrespective of which of the two variables was the non-dichotomous dependent variable), 5 included at least one of the "ratio variables" ("brain mass/body mass" or "blood 5-HT concentration/blood tryptophan concentration"). This result is expected since the ratio of two normally distributed variables is not normally distributed and can even be bimodal [59]. Based on these results, the Mann-Whitney test was used to compare the "ratio variables" in the two sex groups; for all other variables, Student's *t*-test was used. Likewise, Spearman's correlation coefficient was used to correlate two variables when either of them was a "ratio variable"; otherwise, Pearson's correlation was used. The results of these calculations are given in Table 1.

In the interpretation of the results, the Bonferroni correction for multiple tests was used. The type I error (*α *= 0.05) was divided by the total number of tests (36; Table 1) to yield the significance level of α* = 0.0013. As expected, highly significant positive correlations were found between body mass and brain mass (*p *< 10^-6^) and between body mass and gut length (*p *= 10^-5^). Also, a highly significant negative correlation was found between body mass and the brain/body mass ratio (*p *< 10^-6^), possibly due to allometric scaling of brain size.

**Figure 3:**
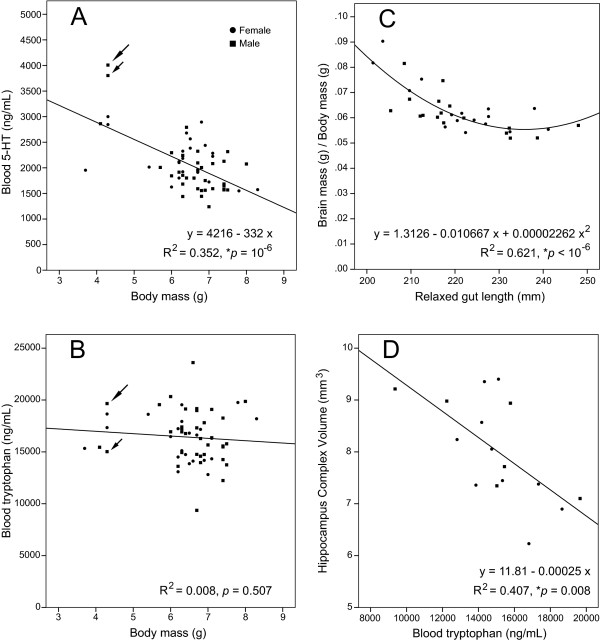
**Relationships between highly correlated variables**. (**A**) The relationship between body mass and blood 5-HT concentration. The long and short arrows indicate two potential outliers with high blood 5-HT concentration. (**B**) The blood tryptophan concentration of the two potential outliers in (A) appears to be normal (the corresponding arrows). (**C**) The relationship between gut length and the brain/body mass ratio. (**D**) The relationship between blood tryptophan concentration and the volume of the hippocampal complex.

The cross-correlational analysis revealed a strong negative correlation between body mass and blood 5-HT levels (*p *= 10^-6^). Also, significant correlations were found between brain mass and blood 5-HT levels, and between the brain/body mass ratio and blood 5-HT levels (*p *= 0.001 and 0.0002, respectively). Since body mass, brain mass and the brain/body mass ratio cross-correlate, we next tested which of the three variables or their linear combinations could best predict blood 5-HT levels by using stepwise (backward) linear regression. It revealed that body mass, alone, could best predict blood 5-HT levels (Fig. 3A) and that inclusion of the other two variables did not statistically improve this prediction. This model yielded *R*^2 ^= 0.352 (*F*_1,56 _= 30.5; *p *= 10^-6^; adjusted *R*^2 ^= 0.341); the residuals were normally distributed (*p *= 0.21) and independent (DW = 1.15). Since two males had unusually high blood 5-HT concentration (over 3500 ng/mL), we next investigated the origin of these outliers and their influence on the regression model. No blood clots were observed in these samples (all cases with blood clots had been eliminated prior to the analysis) and the tryptophan concentration measured in these same samples was in the normal range (Fig. 3B). Since Cook's distances (Cook's *D*) can be used to estimate the influence of individual cases on a regression model, we calculated this statistic for all regression points and found the largest Cook's *D *to be 0.66, below the critical value of 1. While the two cases could not be eliminated based on any of these objective criteria, we next excluded these two cases and recalculated the regression model. After this adjustment, blood 5-HT concentration was still best predicted by body mass (*R*^2 ^= 0.210; *F*_1,54 _= 14.3; *p *= 0.00039; adjusted *R*^2 ^= 0.195).

**Figure 4:**
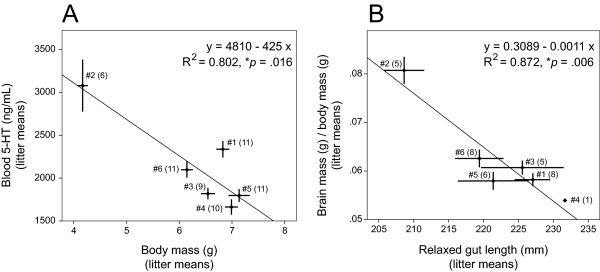
**Relationships between the litter means of highly correlated variables**. (**A**) The relationship between the litter means of body mass and blood 5-HT concentration. (**B**) The relationship between the litter means of gut length and the brain/body mass ratio. In (**A**) and (**B**), point #N (M) represents litter N with M cases analyzed; the horizontal and vertical bars represent the standard errors of the means.

All pups with body mass less than 5 g came from the same litter. Their tissues were collected at PD14, the same developmental time at which the other four litters were processed. The litter was normal size (14 pups; another litter had the same number of pups) and the pups showed no gross anatomical abnormalities. These pups were used in all the other analyses (including the reconstruction of the hippocampal complex) and did not appear to be outliers there (Fig. 3C, D). The correlation between body mass and blood 5-HT remained significant if only the litter means were considered (Fig. 4A).

Next, we analyzed the strong correlation between gut length and the brain/body mass ratio (*p *= 10^-6^). Since the residuals of the linear regression between these two variables were not distributed normally (*p *= 0.03), we modeled their relationship with a quadratic polynomial (Fig. 3C). The regression yielded *R*^2 ^= 0.621 (*F*_2,30 _= 24.6, *p *< 10^-6^; adjusted *R*^2 ^= 0.596); the residuals were normally distributed (*p *= 0.26) and independent (DW = 1.16). The constant at the quadratic term was significantly different from zero (*t*(30) = 3.36, *p *= 0.002), further supporting the non-linearity of the relationship. The correlation between gut length and the brain/body mass ratio remained significant if only the litter means were considered (Fig. 4B).

The correlation between blood tryptophan concentration and hippocampal complex volume did not meet the significance criterion after the Bonferroni correction. However, it was highly significant considered separately (Fig. 3D). The linear regression yielded *R*^2 ^= 0.407 (*F*_1,14 _= 9.63, *p *= 0.008; adjusted *R*^2 ^= 0.365); the residuals were normally distributed (*p *= 0.58) and independent (DW = 1.40).

## Discussion

To date, relationships among body mass, brain mass, gut length, and whole-blood tryptophan and 5-HT levels have not been systematically investigated in a genetically uniform population of any mammalian species. Table 1 provides information for future top-down [2,25] and bottom-up (e.g., structural equation modeling) studies and can also be used in the design of multidimensional experiments where *a priori *considerations of statistical power are important.

We found a strong negative correlation between body mass and whole-blood 5-HT levels. The strength of this relationship was influenced by the presence of several pups with low body mass but no gross anatomical abnormalities. The elevated blood 5-HT levels were unlikely to be caused by developmental delay, since blood 5-HT levels increase, not decrease, during postnatal development in mice [1]. It has been reported that extremely low levels of maternal peripheral 5-HT result in smaller mouse embryos that also exhibit abnormalities in many organ systems [60,61]. It is intriguing to speculate that a less severe decrease in maternal peripheral 5-HT may also result in reduced body mass and compensatory overproduction of peripheral 5-HT in the offspring. However, peripheral administration of the immediate 5-HT precursor 5-hydroxytryptophan (5-HTP) to pregnant rats also leads to embryos with reduced body mass [62].

The observed relationship between body mass and blood 5-HT levels is predicted by our theoretical model of platelet 5-HT levels [2]. According to the model (equations (22) and (27) of [2]), platelet 5-HT levels (*P*) are given by the following equation:

*P *= [*a Q *(*b *+ *Ω*_*g*_^-1^) + *c*]^-1^,

where *Q *is the total cardiac output, *Ω*_*g *_is the volume of the gut wall, and where the positive *a*, *b*, and *c *can be considered constant in this study. The cardiac output is likely to be directly proportional to body mass (i.e., *Q *= *kM*, where *M *is body mass and *k *is a positive constant). Gut length also scales with body mass (Table 1), but an increase in body mass is unlikely to increase gut length by the same factor. Therefore, the volume of the gut wall may scale as *Ω*_*g*_*= rM *^(1/h) ^+ *s*, where *r *> 0, *s *≥ 0, and *h *≥ 1 are constants. This allows expressing *P *as a function of *M *and, since the derivative of *P *with respect to *M *is always negative (i.e., *dP*/*dM *< 0), platelet 5-HT concentration should decrease as body mass increases. Importantly, this relationship may not hold true for developmental processes [1] because other parameters, here considered constant (e.g., the levels of extracellular 5-HT in the gut wall, the platelet 5-HT uptake rate constant; see Janušonis (2008)), may undergo developmental changes.

Body mass is almost never controlled for in experimental studies on the platelet hyperserotonemia of autism [9,11,12]. In light of the present findings, including body mass as a covariate may reduce uncontrolled variability in platelet 5-HT levels. Recent findings show that low birth mass may carry an increased risk for ASDs [63] and that head circumference strongly correlates with body mass in ASD patients [64]. Intriguingly, the maturation of thalamocortical projections appears to be influenced by body mass in normal mice [65].

We found a non-linear relationship between gut length and the brain/body mass ratio. While there appears to be little association between the brain/body mass ratio and the relaxed gut length if guts are average length, short guts predict a higher brain/body mass ratio (Fig. 3C). The biological nature of this relationship is unclear. It is conceivable that the length of the gut modulates how much tryptophan reaches the systemic blood circulation and, since tryptophan can cross the BBB, its availability in the brain may affect brain growth through 5-HT synthesis [66]. Intriguingly, it has been recently hypothesized that a decrease in gut length has had a major impact on the evolution of the human brain [67,68].

The analysis also suggested a tentative relationship between blood tryptophan levels and the volume of the developing hippocampal complex. Tryptophan depletion is known to affect the development and function of the hippocampus [69-71]. Our results suggest that even relatively small, natural fluctuations of blood tryptophan levels may modulate its size. Interestingly, at PD14, the mean blood tryptophan concentration in the CD-1 strain (16424 ng/mL) was considerably higher than that in the C57BL/6 strain (11563 ng/mL) [1]. Since the tryptophan levels were assessed by the same method in both studies, a statistical comparison between them suggests that this difference between the two strains is significant (*t*(66) = 6.17, *p *= 4.7·10^-8^). It would be interesting to know if the mean volumes of the hippocampal complex also differ in these strains.

No sex differences were observed with regard to all variables. The marginally significant sex difference between the blood 5-HT/tryptophan ratios could be spurious considering the large number of tests. Also, the correlation between blood tryptophan and 5-HT was non-significant, consistent with previous reports in humans [34] and in C57BL/6 mice at postnatal day 14 [1].

The strong statistical relationships found in the present study should be cross-validated in other mouse strains and (when feasible) in humans. It should be noted that all of the reported relationships are empirical and do not imply direct causality; however, these findings may stimulate further experimental research where causality in addressed. For instance, mice lacking the serotonin 5-HT_1A _receptor show elevated levels of anxiety [72] and develop an autistic-like blood hyperserotonemia [1]. While these two phenomena may appear unrelated, they may be explained by the expression of 5-HT_1A _receptors both in the brain [51,73,74] and in the gut [75]. Serotonin 5-HT_4 _receptors play important roles in the developing brain [76,77] and the gut [44,78], and recently 5-HT_4 _mRNA has been reported in blood platelets [79]. Therefore, altered expression of 5-HT_4 _receptors may simultaneously affect all of these subsystems, even if they are separated by the BBB. Experimental methods, used in combination with modern statistical approaches, can provide important information about the interactions among components of the peripheral and central 5-HT systems, which are often studied in isolation.

## Conclusion

The study provides information on the covariance structure of several variables related to the central and peripheral serotonin systems. In particular, the results indicate that body mass should be included as a covariate in studies on platelet 5-HT levels and they also suggest a link between brain growth and gut length.

## Authors' contributions

RA, AC, and MT performed the brain and gut analyses; AC genotyped the pups; GMA performed the blood HPLC analysis; SJ, RA, and GMA analyzed the results; SJ conceived the project, supervised all of its stages, and wrote the manuscript.

## Supplementary Material

Additional file 1 **The data set used in the study.** Click here for file
